# IL-22 alters gut microbiota composition and function to increase aryl hydrocarbon receptor activity in mice and humans

**DOI:** 10.1186/s40168-023-01486-1

**Published:** 2023-03-09

**Authors:** Jordan S. Mar, Naruhisa Ota, Nick D. Pokorzynski, Yutian Peng, Allan Jaochico, Dewakar Sangaraju, Elizabeth Skippington, Annemarie N. Lekkerkerker, Michael E. Rothenberg, Man-Wah Tan, Tangsheng Yi, Mary E. Keir

**Affiliations:** 1 Genentech, 1 DNA Way, South San Francisco, CA 94080 USA; 2 Present address: Biomarker Discovery OMNI, Genentech Inc., South San Francisco, CA USA; 3 Department of Infectious Diseases, Genentech Inc., South San Francisco, CA USA; 4 Drug Metabolism and Pharmacokinetics, Genentech Inc., South San Francisco, CA USA; 5 Bioinformatics, Genentech Inc., South San Francisco, CA USA; 6 OMNI Biomarker Development, Genentech Inc., South San Francisco, CA USA; 7 Early Clinical Development, Genentech Inc., South San Francisco, CA USA; 8 Present address: Department of Immunology Discovery, Genentech Inc., South San Francisco, CA USA

**Keywords:** IL-22, Colitis, Inflammatory bowel disease, Aryl hydrocarbon receptor, Tryptophan, Indole, Gastrointestinal microbiome

## Abstract

**Background:**

IL-22 is induced by aryl hydrocarbon receptor (AhR) signaling and plays a critical role in gastrointestinal barrier function through effects on antimicrobial protein production, mucus secretion, and epithelial cell differentiation and proliferation, giving it the potential to modulate the microbiome through these direct and indirect effects. Furthermore, the microbiome can in turn influence IL-22 production through the synthesis of L-tryptophan (L-Trp)-derived AhR ligands, creating the prospect of a host-microbiome feedback loop. We evaluated the impact IL-22 may have on the gut microbiome and its ability to activate host AhR signaling by observing changes in gut microbiome composition, function, and AhR ligand production following exogenous IL-22 treatment in both mice and humans.

**Results:**

Microbiome alterations were observed across the gastrointestinal tract of IL-22-treated mice, accompanied by an increased microbial functional capacity for L-Trp metabolism. Bacterially derived indole derivatives were increased in stool from IL-22-treated mice and correlated with increased fecal AhR activity. In humans, reduced fecal concentrations of indole derivatives in ulcerative colitis (UC) patients compared to healthy volunteers were accompanied by a trend towards reduced fecal AhR activity. Following exogenous IL-22 treatment in UC patients, both fecal AhR activity and concentrations of indole derivatives increased over time compared to placebo-treated UC patients.

**Conclusions:**

Overall, our findings indicate IL-22 shapes gut microbiome composition and function, which leads to increased AhR signaling and suggests exogenous IL-22 modulation of the microbiome may have functional significance in a disease setting.

Video Abstract

**Supplementary Information:**

The online version contains supplementary material available at 10.1186/s40168-023-01486-1.

## Background

Cytokines are secreted proteins that mediate cell–cell communication between immune cells and play key roles in activating pathways involved in immunity and host response to microbes. IL-22, a cytokine secreted by T cells and type 3 innate lymphoid cells (ILC3s) [1, 2], has shown a unique capacity among cytokines to signal to and regulate epithelial cells, inducing a broad range of responses. IL-22 is produced by T cells and ILC3s in response to IL-23 secreted by activated dendritic cells and macrophages [3]. IL-22 signals through its cognate receptor, a heterodimer of IL-10R2 and IL-22RA1, which is predominantly expressed on epithelial cells lining mucosal barriers [4, 5]. Restricted expression of IL-22RA1 effectively targets IL-22 activity to mucosal barriers comprising the primary sites of host-microbe interactions in the skin, airway epithelium, and gastrointestinal (GI) tract.

Through its effects on epithelial cells, IL-22 may regulate microbiome composition and function via both direct and indirect mechanisms. Upon IL-22 binding, intestinal epithelial cells (IECs) upregulate proliferative and anti-apoptotic pathways, antimicrobial protein (AMP) secretion, and mucin production and fucosylation [6]. IL-22-induced AMP secretion by IECs has the potential to cull select bacterial clades, acting as a negative selective pressure on the microbiota. In a mouse model of GI infection, robust IL-22-induced AMP activity provided protection against *Citrobacter rodentium* infection [7]. Conversely, IL-22-regulated mucosal glycosylation selects for commensal organisms that drive colonization resistance to *Clostridioides difficile* [8]. This suggests increases in mucin availability and altered fucosylation downstream of IL-22 signaling may select for microbes capable of metabolizing these host-derived sugars, acting as a positive selective pressure.

Even in the absence of pathogens, homeostatic IL-22 activity may contribute to microbiome composition. Deletion of IL-23, the IL-23 receptor, or IL-22 itself all result in altered gut microbiome composition and increased sensitivity to chemically induced colitis in mice [9–12]. While exogenous administration of IL-22 ameliorates﻿ this phenotype, transferring the microbiome of IL-22 or IL-23 pathway-deficient animals to wild-type mice leads to reduced AMP and IL-22 expression in the gut and, again, increased sensitivity to chemically induced colitis [9, 12, 13]. In sum, IL-22 may play a role in shaping the gut microbiome to exclude potentially pathogenic or pro-inflammatory organisms that may contribute to GI inflammatory diseases such as ulcerative colitis (UC), a type of inflammatory bowel disease (IBD) that is characterized by inflammation of the intestinal mucosa. Patients with UC have been shown to have reduced microbial diversity broadly characterized by reduced microbial richness and uneven abundance distribution in comparison to healthy subjects [14].

Microbes can influence the host immune system through the effects of secreted metabolites or modification of host-derived factors [15, 16]. A classic example of this are short-chain fatty acids (SCFA), which are derived exclusively from microbial fermentation and can regulate colonic regulatory T cell (Treg) numbers; inhibit NF-κB activation in lamina propria cells and peripheral blood mononuclear cells; and enhance antimicrobial activity of intestinal macrophages [17–19]. More recently, microbial metabolism of L-tryptophan (L-Trp), specifically the production of indole derivatives, has been identified for its role in generating ligands for the aryl hydrocarbon receptor (AhR). AhR is a ligand-activated transcription factor that regulates a number of immune processes, including the development, maintenance, and function of ILC3s, γδ T cells, Th22 cells, and Th17 cells [20]. Mice with reduced AhR activity display increased sensitivity to colitis, intestinal candidiasis, and alcoholic liver disease [13, 21–23]. Supplementation with L-Trp-metabolizing microbes or microbially derived L-Trp metabolites, such as indole derivatives, rescues this phenotype [13, 21–23]. Reductions in both SCFA and tryptophan metabolites have been described in IBD patients [13, 24].

Given its activity at the mucosal barrier, IL-22 may act as a critical node for host-microbe communication through its role as a downstream target of ligand-bound AhR. Multiple AhR transcription factor binding sites are found in the IL-22 locus, allowing AhR to regulate the development of ILC3s as well as IL-22 gene expression [6, 20, 25]. Additionally, AhR-deficient animals produce significantly less IL-22 [25]. This suggests AhR can act as a microbial sensor of sorts; detecting products of microbial metabolism and directing the host immune system to respond in kind [20]. Loss of IL-22 is associated with altered microbiome composition and function as well as reduced AhR activation, suggesting potential involvement in a host-microbe feedback loop in the gut that, combined, may increase susceptibility to colitis [12, 13]. Products of microbial metabolism can activate AhR and upregulate IL-22 production, leading to increased AMP and mucin production in IECs which in turn contribute to reduced colitis susceptibility and may shape microbiome composition and function [6].

To address the potential role of IL-22 in this putative feedback loop and in UC, we evaluated the effect of exogenous IL-22, in the form of a fusion protein of IL-22 and the Fc portion of IgG4 (IL-22Fc), on the GI microbiome, microbial L-Trp metabolism, and AhR activity in mice and UC patients, a disease associated with dysbiosis, altered microbial L-Trp metabolism, and reduced AhR activity in the GI tract [13, 21, 26–28]. In doing so, we observed an IL-22Fc-induced shift in microbiota composition and microbial L-Trp metabolism that led to increased fecal AhR activity in both mice and UC patients treated with IL-22Fc.

## Results

### IL-22Fc treatment alters microbiota composition along the GI tract in mice

**Fig. 1:**
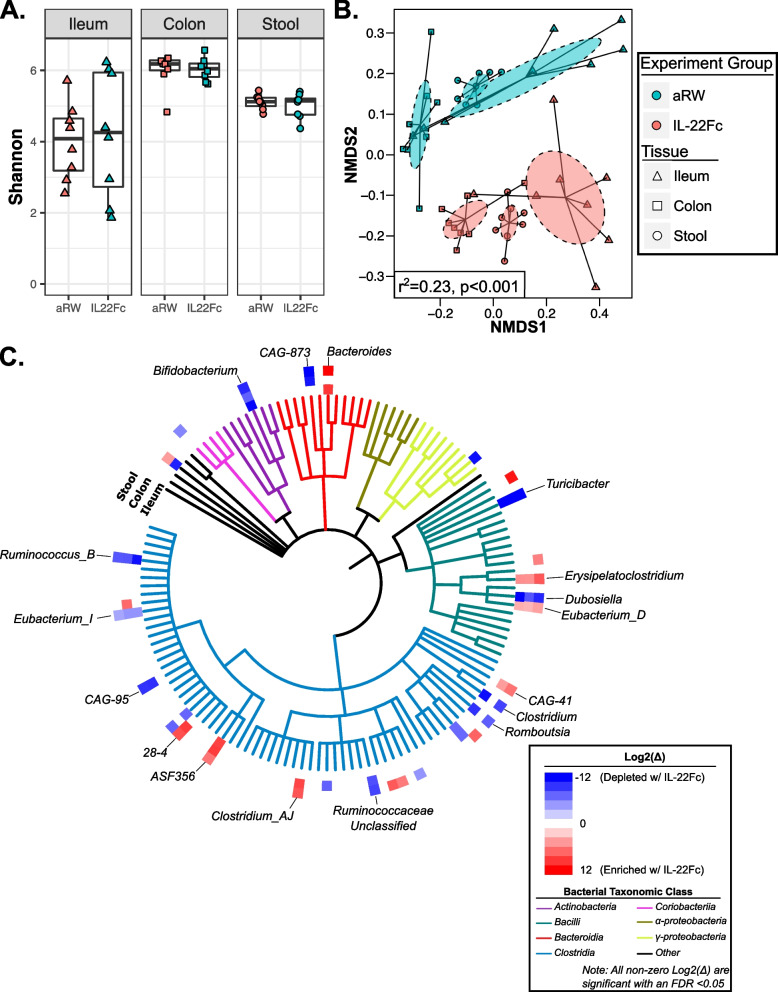
IL-22Fc consistently alters microbiota composition along the GI tract in mice. **A** Shannon’s diversity following IL-22Fc or aRW treatment. **B** NMDS ordination plot of Bray-Curtis distances. Dashed ellipses represent the 95% CI for the centroid of each stratification group. *r*^2^ calculated by PERMANOVA. For **A** and **B**, triangle, square, and circle markers indicate ileal, colonic, and fecal samples, respectively. Teal and red markers indicate animals treated with IL-22Fc (*n*=8) or isotype control (*n*=8), respectively. **C** Taxonomic tree depicting all bacterial genera detected within the ileum, colon, or stool of IL-22Fc or aRW-treated mice. The outer heatmap displays the difference in mean, normalized abundance of a given bacterial genera between IL-22Fc and aRW-treated mice (Log_2_(*∆*)) in the ileum, colon, or stool. All non-zero Log_2_(*∆*) values have a BH-adjusted *p* value <0.05. Highly IL-22Fc responsive genera, defined as the top ten genera based on average Log_2_(*∆*) and significant enrichment or depletion in IL-22Fc-treated animals across multiple tissues, are labeled. Note that only seven IL-22Fc-enriched genera met these criteria

To assess the impact of IL-22Fc treatment on the GI microbiome, previously co-housed wild-type adult mice were randomly re-caged into two, separately housed treatment groups, and injected intraperitoneally with either IL-22Fc or anti-Ragweed (aRW, isotype control antibody) (Fig S1A, see the “Methods” section). After 2 weeks of treatment, animals were euthanized and ileal, colonic, and fecal samples were collected for DNA isolation and subsequent 16S-V4 rRNA gene sequencing to characterize the bacterial microbiota. Shannon’s diversity was unaltered following IL-22Fc treatment (Fig. 1A), though a trend towards reduced richness was observed (Fig. S1B). Analysis of Bray-Curtis distances revealed a significant compositional shift in the bacterial microbiota of IL-22Fc-treated mice compared to isotype-treated animals across all three GI regions while maintaining the relative biogeographical structure of these tissue-specific communities (Fig. 1B).

To identify highly IL-22Fc responsive genera, we first identified genera that were significantly enriched (or depleted) in multiple tissues of IL-22Fc treated mice (i.e., ileum, colon, and/or stool) based on differential abundance (DA) testing utilizing DESeq2 [29] (Fig. 1C, Table S1-3). We then ranked these multi-tissue enriched (or depleted) genera by their average difference in normalized abundance between IL-22Fc and aRW-treated mice (Log2(*∆*)) across these tissues to identify those with the greatest effect size, designating the top ten genera based on this ranking as highly IL-22Fc enriched (or depleted). Based on this criteria, seven genera were identified as highly IL-22Fc enriched (only seven genera were significantly enriched in multiple tissues): *Bacteroides, 28-4* (a member of the *Lachnospiraceae* family)*, Clostridium-AJ, Altered Schaedler Flora 356* (*ASF-356*)*, Erysipelatoclostridium, CAG-41* (a poorly characterized member of the *Clostridia* class), and *Eubacterium-D*, suggesting IL-22Fc treatment creates a more permissive environment that allows these organisms to thrive (Fig. 1C and S1C). Conversely, based on the above criteria, *Turicibacter, CAG-873* (a member of the *Muribaculaceae* family), *Dubosiella, Bifidobacterium, CAG-95* (a member of the *Lachnospiraceae* family), *Clostridium*, *Ruminococcus-B,* an unclassified genus within the *Ruminococcaceae* family (*Ruminococcaceae unclassified), Romboutsia, and Eubacterium-I* represented the top ten genera that were highly depleted following IL-22Fc treatment (Figs. 1C and S1D). To ensure these observations were robust to the statistical method implemented for the identification of DA genera and not an artifact of the DESeq2 approach, we performed two additional, conceptually different statistical approaches for DA testing: nonparametric Wilcoxon rank-sum testing as well as compositional data analysis utilizing ALDEx2 [30] as recommended by Nearing et al. (see the “Methods” section) [31]. When comparing the statistical results of DESeq2, Wilcoxon rank-sum, and ALDEx2, significant DA genera (BH-corrected *p* value <0.05) were consistently identified across the three approaches (Table S1-3). In a minority of genera that were only identified as significant by DESeq2, they frequently were nominally significant based on Wilcoxon rank-sum and/or ALDEx2 analysis (uncorrected *p* value <0.05) and routinely demonstrated small effect sizes compared to multi-significant genera. Combined, these findings demonstrate the robustness of our observations and confirm IL-22 as a robust microbiome modulator capable of altering the steady-state microbial community along the GI tract of healthy mice.

### IL-22Fc treatment alters microbial tryptophan metabolism

**Fig. 2:**
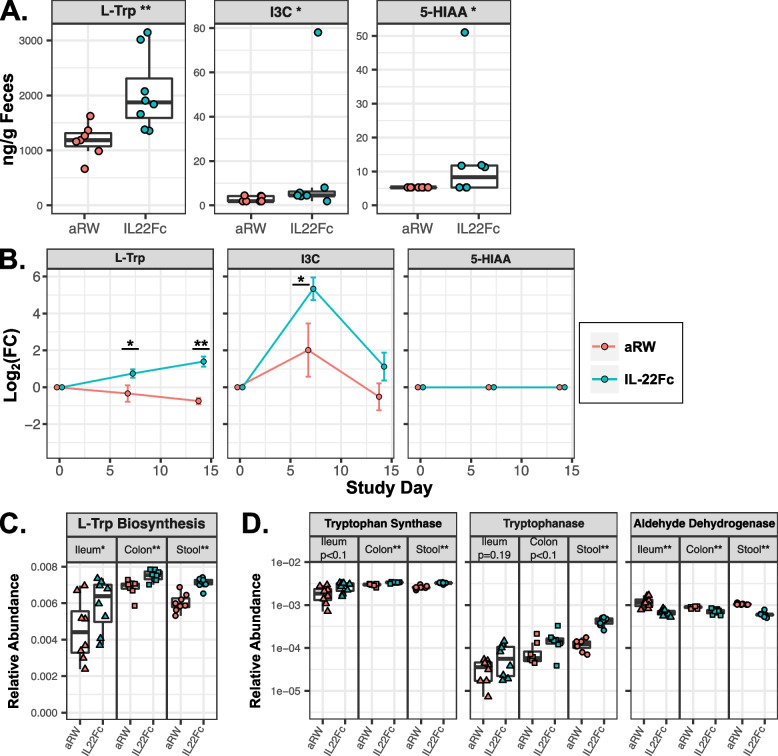
IL-22Fc alters microbial tryptophan metabolism in mice. **A** Fecal concentrations of L-Trp, I3C, and 5-HIAA following IL-22Fc or aRW treatment. **B** Log_2_ fold change from baseline in fecal concentrations of L-Trp, I3C, and 5-HIAA. **C** Predicted metagenomic counts, as calculated by PICRUSt2, mapping to the L-tryptophan biosynthesis pathway in IL-22Fc or aRW-treated animals. **D** Predicted metagenomic counts, as calculated by PICRUSt2, of microbial enzymes capable of synthesizing tryptophan (tryptophan synthase), indole (tryptophanase), and 5-HIAA (aldehyde dehydrogenase). Statistical significance determined by rank-sum test (*p<0.05, **p<0.01). Triangle, square, and circle markers indicate ileal, colonic, and fecal samples, respectively. Teal and red markers indicate animals treated with IL-22Fc (*n*=8) or isotype control (*n*=8), respectively

L-Trp and indole derivatives of L-Trp are capable of shaping the intestinal environment﻿ via activation of AhR [20]. Given the reciprocal regulation of L-Trp metabolism and IL-22 production, we initially utilized additional stool samples collected at the same time from the same mouse experiment as stool samples used for microbiota profiling (i.e., after 2 weeks of either aRW or IL-22Fc treatment, Fig. S1A) to assess changes in fecal concentrations of microbially derived L-Trp metabolites following IL-22Fc treatment (see the “Methods” section). Fecal pellets from IL-22Fc-treated animals had increased concentrations of L-Trp, indole-3-carboxaldehyde (I3C), an established AhR ligand [20], and 5-hydroxyindoleacetate (5-HIAA) compared to controls (Fig. 2A). The remaining microbially derived L-Trp metabolites in our panel were unchanged (Fig. S3A). Of note, a previous study describing the role of microbially derived L-Trp derivatives in the context of Card9 deficiency did not observe an enrichment of tryptamine (a potential precursor to indole derivative synthesis) in animals expressing higher levels of IL-22 nor were fecal tryptamine concentrations associated with IBD status in humans [13]. This suggests that increased synthesis of indole derivatives proceeds through an alternative precursor, such as indole pyruvate, or changes in fecal tryptamine concentrations following increased IL-22 levels are undetectable due to rapid metabolic pathway kinetics quickly converting excess tryptamine to downstream products. In a separate replicate murine experiment, stool samples were collected at days 0, 8, and 14 to longitudinally assess changes in fecal concentrations of microbially derived L-Trp metabolites after IL-22Fc exposure compared to baseline pre-exposure levels. L-Trp, I3C, and two additional microbially derived AhR ligands, indole-3-propionic acid (IPA) and indole-3-acetic acid (IAA) [20], showed a greater increase from the baseline in IL-22Fc-treated animals compared to control-treated animals (Figs. 2B and S3B). The remaining microbially derived L-Trp metabolites (including 5-HIAA, which was not detected in this experiment) showed no difference between IL-22Fc and control treated animals. L-Trp is an essential amino acid obtained via diet or microbial synthesis while I3C, IPA, and IAA are predominantly synthesized by the microbiome via the metabolism of L-Trp [20, 32, 33] (Fig. S2A). Functional analysis of the microbiome utilizing PICRUSt2, a software tool developed for predicting functional abundances based on 16S rRNA gene sequencing﻿ [34], showed predicted metagenomic counts mapping to the L-Trp biosynthesis pathway were significantly increased following IL-22Fc treatment (Fig. 2C). Consistent with this observation, the predicted metagenomic counts of both tryptophan synthase and tryptophanase, enzymes responsible for the synthesis of L-Trp and indole derivatives such as I3C [33], were also increased throughout the GI tract following IL-22Fc treatment (Fig. 2D) though tryptophan decarboxylase, indole pyruvate decarboxylase, and monoamine oxidase (additional enzymes involved in the synthesis of indole derivatives) were not called by PICRUSt2 prediction. Furthermore, a greater proportion of the Amplicon Sequence Variants (ASVs) representing the highly IL-22Fc enriched genera from our 16S-V4 rRNA gene sequencing data were predicted to encode tryptophan synthase compared to ASVs representing the highly IL-22Fc depleted genera while tryptophanase prevalence did not significantly differ (Fig. S3C). Specifically, the predicted prevalence of tryptophan synthase was observed in all ASVs representing the highly IL-22Fc-enriched genera except for those of *Eubacterium-D* (Fig. S3D), the genus with the lowest overall abundance and effect size among the highly IL-22Fc enriched genera (Fig. S1C). While only ASVs representing *Romboutsia* and *Bacteroides* were predicted to encode tryptophanase (Fig. S3D), the high overall abundance of *Bacteroides* compared to *Romboutsia* may indicate an amplified ability of *Bacteroides* to influence concentrations of indole derivatives such as I3C in vivo, leading to the observed increase in fecal concentrations of I3C (Fig. 2A, B). 5-HIAA, while predominately a product of host serotonin metabolism, can also be synthesized by select microbiota members [35, 36] (Fig. S2A). Predicted metagenomic counts of aldehyde dehydrogenase, responsible for 5-HIAA synthesis, decreased with IL-22Fc treatment and the prevalence of this enzyme did not differ significantly between ASVs representing highly IL-22Fc-enriched and depleted genera (Figs. 2D and S2C-D), indicating IL-22Fc driven shifts in the microbiota were not directly responsible for the observed increase in fecal 5-HIAA concentration. Combined, these observations suggest IL-22Fc exposure alters microbial L-Trp metabolism in the gut, resulting in increased concentrations of L-Trp and indole derivatives.

### Fecal AhR activity increases following IL-22Fc treatment

**Fig. 3:**
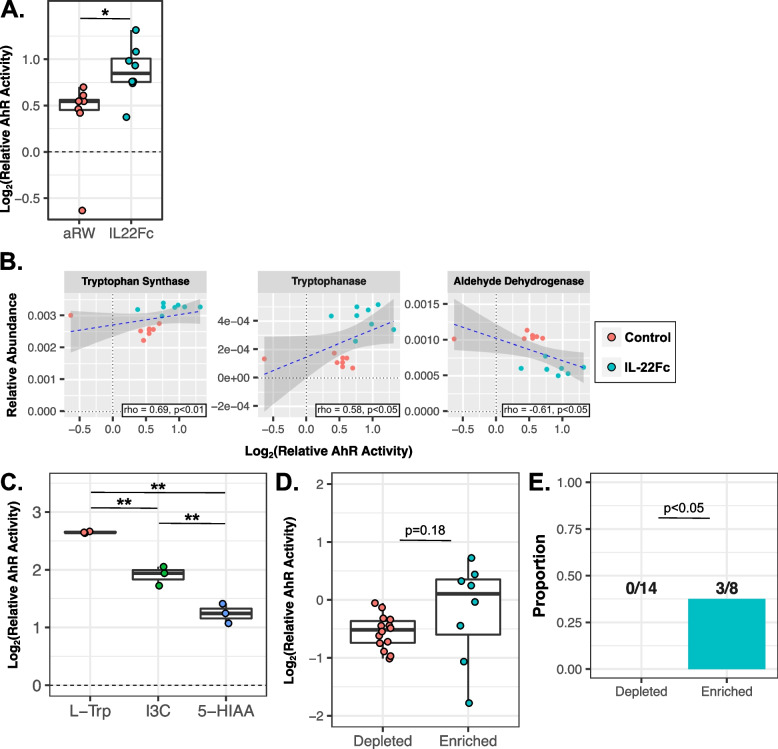
Microbially derived AhR ligand availability in the intestine increases following IL-22Fc treatment in mice. **A** AhR activity following treatment with prepared fecal samples obtained from IL-22Fc or aRW-treated animals, reported as Log_2_(fold change over unstimulated wells). **B** Scatterplot of predicted fecal metagenomic counts of tryptophan synthase, tryptophanase, and aldehyde dehydrogenase vs. fecal AhR activity. Spearman’s rank correlation coefficient (rho) was calculated to determine correlation strength. **C** AhR activity, reported as Log_2_(fold change over unstimulated wells), following treatment with 100uM of either L-Trp, I3C, or 5-HIAA (see the “Methods” section). **D** AhR activity following treatment with sterile-filtered bacterial culture supernatant from bacterial isolates representing either highly IL-22Fc depleted (*n*=14) or enriched (*n*=8) genera, reported as Log2(fold change over matched sterile culture media). **E** Proportion of bacterial isolates representing highly IL-22Fc-depleted or -enriched genera capable of activating AhR in vitro, as defined by an in vitro AhR activity statistically greater than that of matched sterile culture media (see Fig. S5). For A and B, teal and red markers indicate animals treated with IL-22Fc (*n*=8) or isotype control (*n*=8), respectively. For panels **A**, **C**, and **D**, all samples were measured in triplicate and statistical significance was determined by *T* test. For panel **E**, the statistical significance was determined by Barnard’s test. For all panels, *p<0.05 and **p<0.01

IL-22Fc treatment modulates the microbiome in mice, which may indirectly influence host activity through elevated AhR ligand production, including L-Trp metabolites. We next evaluated the broader effects of IL-22Fc and associated microbiome changes on AhR activity using a well-characterized in vitro AhR reporter cell line [37]. AhR activity was subsequently compared with metabolomic and predicted metagenomic results. Fecal pellets from IL-22Fc-treated animals displayed increased AhR activation compared to the control group (Fig. 3A), indicating an IL-22Fc-associated increase in AhR ligands consistent with our metabolomic findings. Despite fecal AhR activity strongly correlating with fecal concentrations of L-Trp, I3C, and 5-HIAA (Fig. S4A), only predicted metagenomic counts of tryptophan synthase and tryptophanase positively correlated with AhR activity, regardless of GI location (Figs. 3B and S4B-C), suggesting microbially derived L-Trp and I3C (rather than 5-HIAA) is driving increased AhR activity associated with IL-22Fc treatment. In support of this, exposing the AhR reporter cell line to 100uM of L-Trp or I3C led to significantly higher AhR activation compared to 5-HIAA, with L-Trp producing the greatest effect (Fig. 3C).

To further evaluate the potential production of AhR ligands by highly IL-22Fc responsive bacterial genera, we used isolates with ≥97% 16S rRNA gene sequence similarity to that of ASVs representing the highly IL-22Fc responsive genera identified earlier and assessed their capacity for AhR ligand production in vitro using the same reporter cell line as above. Strains representing the *Bifidobacterium* (*n*=3), *CAG-873* (*n*=1), *Clostridium* (*n*=2), *Romboutsia* (*n*=1), *Dubosiella* (*n*=1), *Turicibacter* (*n*=1), *Bacteroides* (*n*=3), *Clostridium-AJ* (*n*=3), and *Erysipelatoclostridium* (*n*=1) genera were obtained from public bacterial strain collections (Table S4). Additionally, utilizing stool samples collected from the previously described mouse experiments, we isolated additional strains representing *Bifidobacterium* (*n*=1), *CAG-873* (*n*=4), and *Bacteroides* (*n*=1) (Table S4). All told, we obtained fourteen and eight strains representing highly IL-22Fc enriched and depleted genera, respectively. To assess their ability to activate AhR in vitro, all strains were grown in liquid growth media for 48 h after which culture supernatant was collected, sterile filtered, and assessed for AhR activation relative to matched sterile growth media (see the “Methods” sections). Overall, culture supernatant from highly IL-22Fc-enriched strains induced numerically greater AhR activity compared to that of highly IL-22Fc-depleted strains (Fig. 3D). Furthermore, the proportion of highly IL-22Fc-enriched strains with a relative AhR activity significantly greater than that of matched sterile culture media (indicating their ability to activate AhR) was significantly higher than that of highly IL-22Fc-depleted strains, with culture supernatant from one *Bacteroides* strain (GNE6609) and two *Clostridium-AJ* strains (GNE6686, GNE6624) activating AhR above background while no strains representing highly IL-22Fc depleted genera did so (Figs. 3E and S5). Of note, culture supernatant from an additional *Bacteroides* strain (GNE6603) also induced AhR activity compared to matched sterile growth media but this signal was not significant due to an outlier replicate (Fig. S5B). Publicly available whole-genome sequence data for these four AhR activating strains (GNE6609 [Bacteroides thetaiotaomicron VPI-5482; GCF_000011065.1], GNE6603 [Bacteroides faecis MAJ27; GCF_900106755.1], GNE6686 [Ruminococcaceae bacterium D16; GCA_000177015.3], and GNE6624 [Clostridiales bacterium Choco116; GCA_003202955.1]) was analyzed to look for putative presence of tryptophan synthase, tryptophanase, indole pyruvate decarboxylase, tryptophan decarboxylase, monoamine oxidase, and aldehyde dehydrogenase homologs, key bacterial enzymes involved in L-Trp synthesis and subsequent conversion to AhR activating indole derivatives (Fig. S2). Consistent with our predictions based on PICRUSt2 analysis, putative tryptophan synthesis homologs were detected in the genome of all four AhR activating strains and tryptophanase homologs were present in the two AhR activating *Bacteroides* strains (GNE6609 and GNE6603) while tryptophan decarboxylase and monoamine oxidase homologs were not detected in any AhR activating strain (Table S5). Interestingly, putative indole pyruvate decarboxylase homologs were also present in all four AhR activating strains, despite PICRUSt2 predictions not indicating such, and aldehyde dehydrogenase homologs were detected in three of these strains (GNE6609, GNE6686, and GNE6624) (Table S5). Combined, these observations suggest all four AhR activating strains are capable of synthesizing L-Trp and L-Trp-derived AhR ligands. As such, targeted metabolomics was performed on culture supernatants from all four AhR activating strains to assess their ability to synthesize L-Trp-derived AhR ligands in vitro. Culture supernatant collected from the *Clostridium-AJ* strains (GNE6686 and GNE6624) had increased concentrations of IAA, culture supernatant from *Bacteroides* strain GNE6609 had increased levels of L-Trp, and culture supernatant from *Bacteroides* strain GNE6603 had increased levels of both IAA and L-Trp relative to matched sterile growth media (Fig. S5C), indicating these strains can synthesize IAA and/or L-Trp in vitro (consistent with our PICRUSt2 and WGS analysis). All told, these results further indicate that altered L-Trp metabolism associated with IL-22Fc-induced changes in GI microbiome composition leads to increased AhR activity in the gut.

### IL-22Fc administration increases fecal AhR activity in humans

**Fig. 4:**
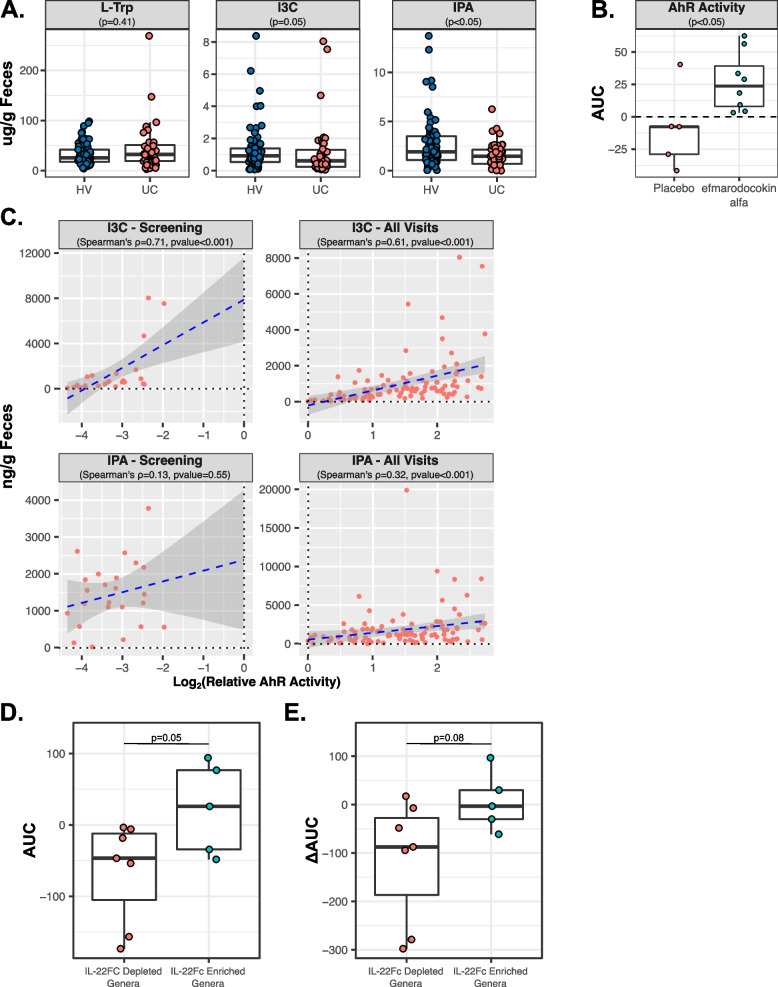
Efmarodocokin alfa administration increases fecal AhR activity in humans. **A** Fecal concentrations of L-Trp, I3C, and IPA at screening in HV (*n*=69) compared to UC (*n*=46). *P* value determined by rank-sum test. **B** AUC of Log_2_(fold change from screening) for fecal AhR activity in enrolled UC patients following administration of efmarodocokin alfa (*n*=8) or placebo (*n*=5). **C** Scatterplot of fecal AhR activity vs. fecal concentrations of I3C and IPA at screening only (*n*=23) and at all visits (*n*=104) in enrolled UC patients. Spearman’s rank correlation coefficient (rho) was calculated to determine correlation strength. **D** Mean AUC of Log_2_(fold change from screening) for normalized abundance of IL-22Fc-depleted (*n*=7) or -enriched (*n*=5) genera detected in enrolled UC patients receiving efmarodocokin alfa treatment. **E** Mean ΔAUC (AUC in efmarodocokin alfa-treated UC patients—AUC in placebo-treated UC patients) of IL-22Fc-depleted (*n*=7) or -enriched (*n*=5) genera detected in enrolled UC patients. For panels **B**, **D**, and **E**, only enrolled UC patients with a complete sample collection history (screening, day 29, day 43, day 64, and day 85) were considered for AUC calculations. *P* value determined by Student’s *T* test

Experiments in mouse models suggested IL-22Fc treatment alters microbiome composition and subsequent AhR ligand production. UC patients have been previously shown to have microbial dysbiosis, altered microbial L-Trp metabolism, and reduced AhR activity and treatment with an exogenous IL-22 pathway is being evaluated as a therapy for UC [13, 21, 26, 28, 38–40]. We next evaluated the effects of IL-22Fc on fecal L-Trp metabolite concentrations, AhR activity, and microbiota composition in UC patients participating in a phase 1b randomized, blinded, placebo-controlled multiple ascending-dose study evaluating the safety, tolerability, pharmacokinetics, and pharmacodynamics of repeat intravenous dosing of efmarodocokin alfa, a fusion protein that links IL-22 with human immunoglobulin G4 (NCT02749630) [41]. At screening, prior to drug administration, fecal levels of the microbially synthesized indole derivatives I3C and IPA were reduced in UC patients compared to healthy volunteers (HV) while fecal L-Trp levels were unchanged (Fig. 4A). This was accompanied by increased fecal concentrations of L-Kynurenine (L-Kyn, Fig. S6A) in UC, suggesting a shunting of L-Trp metabolism towards host pathways. Consistent with these findings, UC stool also elicited reduced AhR activity in our in vitro AhR reporter assay, though this did not meet statistical significance (Fig. S6B). Baseline modified Mayo Clinic Scores (mMCS) were available for the subset of screened UC patients that met the study enrollment criteria (including diagnosis of UC with a Mayo Endoscopic Subscore of ≥2 points by central reading at screening) and were enrolled in the clinical trial [41]. When stratified by mMCS, we observed reduced IPA coupled with significantly increased L-Kyn in severe UC (mMCS >5) compared to moderate UC (mMCS ≤5), with levels of L-Trp and I3C being similar amongst moderate and severe UC (Fig. S6C). Severe UC was also accompanied by reduced fecal AhR activity compared to moderate UC, though this again did not meet statistical significance (Fig. S6D). Additionally, we previously described microbial dysbiosis in the fecal microbiota of enrolled UC participants compared to HV, which was broadly characterized by reduced diversity [41]. Human and mouse microbiota do not completely overlap, and we observed five of the highly IL-22Fc enriched and eight of the highly IL-22Fc depleted genera in the fecal microbiota of enrolled subjects out of the 17 highly IL-22Fc responsive bacterial genera identified in our mouse experiment (Figs. 1C and S1C-D). Of these, the mean Log_2_(fold change in abundance at screening between UC and HV) of the IL-22Fc depleted genera was greater than that of the IL-22Fc enriched genera, indicating IL-22Fc depleted genera tended to be enriched in UC, though this trend was not significant (Fig. S7A). Looking at the individual genera, only *Clostridium*, *Ruminococcaceae unclassified*, and *Eubacterium-D* were significantly enriched in UC participants compared to HV at screening while no genera were significantly depleted (Fig. S7B-C). These observations are in line with prior studies describing depleted indole concentration, increased L-Kyn levels, and reduced AhR activity accompanying microbial dysbiosis in the stool of IBD patients [13, 23, 28].

Following the screening, enrolled subjects were administered either placebo or efmarodocokin alfa according to one of three dosing regimens (see the “Methods” section), and fecal L-Trp metabolite concentrations, AhR activity, and microbiota composition were further evaluated post-treatment. In addition to screening samples, stool samples were collected from enrolled participants on days 29, 43, 64, and 85. Fecal L-Trp metabolite concentrations, AhR activity, and IL-22Fc responsive bacterial genera abundances were assessed in these samples, normalized to screening levels, and the area under the Log_2_(fold change from screening) curve (AUC) was calculated for each participant to determine the impact of efmarodocokin alfa compared to placebo over the duration of treatment. As determined by AUC comparisons, UC participants receiving efmarodocokin alfa showed a statistically significant increase in fecal AhR activity compared to placebo-treated participants that was accompanied by an increase in fecal L-Trp, ICA, and IPA, and reduction in fecal L-Kyn, (Figs. 4B, S6E), suggesting efmarodocokin alfa alters AhR activity and L-Trp metabolism in the GI tract of humans. Furthermore, fecal AhR activation strongly correlated with fecal ICA and, to a lesser extent, fecal IPA concentrations in enrolled UC participants both at screening and when considering all visits (Fig. 4C), consistent with our observations in mice. Clinical remission (defined as attaining a mMCS ≤2, Mayo rectal bleeding (RB) subscore of 0, and other Mayo subscores of ≤1) were observed in 5 out of 18 UC participants treated with efmarodocokin alfa compared to 0 of 6 placebo-treated participants, making comparisons between efmarodocokin alfa remitters and non-remitters difficult due the small group sizes [41].

With respect to the fecal microbiota, we previously described a general correction in UC-dysbiosis following efmarodocokin alfa treatment whereby bacterial genera enriched in UC participants compared to HV at screening tended to be depleted following efmarodocokin alfa treatment and vice versa [41]. We next focused on the subset of IL-22Fc-responsive bacterial genera identified in mice that were also present in UC participants. All of the highly IL-22Fc-depleted genera identified in mice and detected in enrolled UC patients tended to be depleted in efmarodocokin alfa-treated UC participants compared to placebo, based on AUC analysis, while the opposite was true of the IL-22Fc-enriched genera, though to a lesser extent (Fig. S8). While these trends were not statistical significant on an individual genus level, grouping these genera together based on their response in mice (i.e., IL-22Fc depleted vs. enriched) indicated the average AUC of IL-22Fc-depleted genera in efmarodocokin alfa-treated UC patients was significantly less than that of the IL-22Fc-enriched genera (Fig. 4D). Additionally, when taking into account the average AUC of these genera in placebo-treated UC participants to calculate ΔAUC (AUC in efmarodocokin alfa-treated UC patients—AUC in placebo-treated UC patients), the average ΔAUC of IL-22Fc-depleted genera was less than that of IL-22F-enriched genera (Fig. 4E). These observations suggest the IL-22Fc responsiveness of key bacterial genera is conserved across mice and humans and, combined with our fecal metabolite and AhR activity findings, provide evidence that IL-22Fc exposure consistently impacts the microbiome/L-Trp/AhR axis in the gut of both mice and humans, warranting future validation in a large cohort.

## Discussion

The microbiome is a key regulator of IL-22 activity via the synthesis of L-Trp-derived AhR ligands [13, 21, 22, 42, 43]. Subsequent IL-22-driven upregulation of AMP secretion, mucin production, and fucosylation by IECs suggests IL-22 in turn shapes the microbiome. We found that IL-22Fc administration resulted in a significant shift in microbiome composition throughout the GI tract of healthy mice, suggesting exogenous IL-22 treatment is a robust microbiome regulator. IL-22Fc also increased L-Trp and its associated metabolites I3C and 5-HIAA, all of which activated AhR in vitro, as well as overall AhR activity in stool samples. Functional analysis of the microbiome showed an increase in microbial L-Trp metabolism genes following IL-22Fc treatment that correlated with AhR activity. Finally, UC patients treated with efmarodocokin alfa, an IL-22Fc fusion protein under evaluation in the clinic, showed increased AhR activity that correlated with fecal ICA and IPA levels and accompanied changes in fecal microbiome composition consistent with that of IL-22F-treated mice, demonstrating consistent effects of IL-22Fc between mice and humans.

Exogenous IL-22 treatment is currently being investigated in multiple disease settings [44] (NCT02749630, NCT04539470, NCT02833389). Characterizing the impact increased IL-22 activity has on the microbiome is essential to understanding the therapeutic value of exogenous IL-22 treatment, especially in a disease setting such as IBD where microbial dysbiosis is a defining feature [26, 27]. Microbiome modification through specific enrichment and depletion of bacterial taxa could be a key mechanism of efficacy following treatment with exogenous IL-22. A number of the IL-22Fc-enriched taxa identified here were previously described to play beneficial roles in host immune regulation. *ASF356*, *Bacteroides*, *Lachnospiraceae 28-4*, and *Clostridium-AJ* members are robust producers of SCFAs [17, 45–47], microbially derived fermentation products that are associated with increased colonic Treg numbers, inhibition of NF-κB activation, and enhanced antimicrobial activity of intestinal macrophages [17, 45–47]. Furthermore, direct supplementation with certain *Bacteroides* members protects against murine models of colitis, graft-versus-host disease (GVHD), and metabolic syndrome [45, 48, 49]. IL-22Fc-depleted microbes, on the other hand, tend to be associated with pro-inflammatory responses. *Turicibacter*, *Dubosiella*, *Ruminococcus*, and *Muribaculaceae* members are reported to be enriched in both murine and human colitis, metabolic syndrome, and GVHD [43, 50–59], with both *Ruminococcus* and *Muribaculaceae* members directly demonstrating pro-inflammatory effects in vitro and in DSS-induced colitis, respectively [60, 61]. These effects are consistent with observations in UC patients undergoing IL-22Fc treatment as microbes that were differentially abundant in UC patients in comparison to healthy subjects at baseline were preferentially altered following IL-22Fc treatment with microbes enriched in UC compared to healthy subjects tending to be depleted following IL-22Fc treatment (and vice versa) [41]. Together, these results suggest treatment with IL-22 may correct disease-associated dysbiosis via selection for beneficial microbes and against disease associated taxa.

Altering microbial L-Trp metabolism is a mechanism by which IL-22Fc-driven microbiome modulation may be beneficial in a disease setting. Reduced concentrations of microbially synthesized L-Trp metabolites (specifically indole derivatives such as ICA and IPA) were found in UC stool compared to healthy volunteers, consistent with previous studies [13, 21, 28]. Indole derivatives confer protection in murine models of colitis, largely via AhR activation, leading to increased IL-22 production by ILC3s and T cells that drives improved intestinal barrier integrity, reduced inflammation, and, ultimately, disease amelioration [20, 22, 28, 62–64]. In our studies, IL-22Fc treatment increased fecal concentrations of microbially derived ICA and IPA, which correlated with increased fecal AhR activity in both mice and humans. Furthermore, predicted metagenomics, WGS analysis, and in vitro culture of IL-22Fc-enriched microbes indicated these organisms have increased capacity for AhR ligand production. This suggests that, in addition to established direct effects of IL-22Fc on the host, IL-22Fc treatment may also confer additional benefits via a feedback loop along the IL-22/microbiome/AhR axis to induce a robust, sustained response.

Although our data support IL-22 as a key regulator of gut microbiome composition and function resulting in increased AhR activity, more work will need to be done, particularly in human subjects, to confirm these findings. Though we provide evidence of IL-22Fc-induced shifts in microbiome composition, we can only hypothesize this is via increased AMP and mucin secretion and future studies are necessary to determine the specific mechanisms involved. Utilization of co-culture systems and further functional analysis of bacterial clades identified here to be enriched or depleted by IL-22Fc treatment may also provide additional insight. The IL-22Fc responsive bacterial strains examined in this study were grown using the preferred in vitro growth conditions for each organism, which limited full evaluation of the functional capacity of bacteria using different media or substrates. As L-Trp and L-Trp precursor availability may vary depending on growth media, it is possible the ability of each strain to activate AhR may vary with growth conditions as well. It is also worth noting that the in vitro behavior of these select microbes may differ from their behavior within a complex gut ecosystem with respect to L-Trp metabolism and AhR ligand synthesis. Additionally, while the consistency among mouse and human observations presented here strengthens the translatability of our findings, the small study size in humans necessitates further validation in a larger cohort, ideally including a greater longitudinal aspect to better characterize the durability of IL-22Fc-associated changes in the GI microbiome and metabolome. To that end, a larger ongoing phase 2 clinical trial of efmarodocokin alfa may yield samples capable of addressing this question (NCT03558152, NCT03650413).

## Conclusions

Our current work expands on the relationship between the GI microbiome and IL-22 by establishing IL-22 as a clear microbiome modulator in both mice and humans. We observed microbiome modulation, increased fecal concentrations of key indole derivatives, and amplified AhR activity in healthy mice following IL-22Fc treatment. Bacterial genera modulated by IL-22Fc treatment were associated with L-Trp metabolism through functional analysis and generated differential AhR ligand production in vitro. Finally, UC patients were observed to have depleted concentrations of AhR ligands and reduced AhR activity in stool samples in comparison to healthy subjects, both of which increased following efmarodocokin alpha treatment. Our findings support a model in which microbial dysbiosis leading to altered L-Trp metabolism, reduced AhR activation, and insufficient IL-22 levels can promote a vicious cycle fostering a loss of GI homeostasis and suggest that supplemental IL-22 may reverse this process, reshaping the microbiome to increase AhR activity and enhance a virtuous cycle to help regain a homeostatic state.

## Methods

### Murine IL-22Fc treatment experimental design

Six- to eight-week-old, female C57BL/6J mice (*n*=16) were purchased from The Jackson Laboratory and allowed to acclimatize in our facilities for 2 weeks prior to the start of the study. After the acclimatization period during which animals were co-housed, mice were randomly re-caged on study day 0 into two treatment groups and separately housed for the remainder of the experiment: IL-22Fc treated (*n*=8, four mice per cage) and control (*n*=8, four mice per cage). Animals were treated with 50 ug/mouse of either IL-22Fc or anti-ragweed isotype control via intraperitoneal injection three times a week and euthanized on day 14. Immediately prior to euthanization, mice were allowed to defecate directly into sterile microcentrifuge tubes. Collected fecal pellets were snap-frozen on dry ice until able to be stored at –80°C. Following euthanization, the terminal ileum (about 3 cm) and the terminal third of the colon (about 3-cm tissue, starting from the rectal end, minus the anus) were collected from each animal. The luminal contents of ileal and colonic samples were removed by gentle squeezing with the forceps, and the tissue was preserved in RNAlater at 4°C overnight and then stored at –80°C. All animal experiments were approved by the Genentech Institutional Animal Care and Use Committee.

### DNA extraction

DNA was extracted from ileal, colonic, and fecal samples using the QIAGEN Allprep DNA/RNA 96 Kit (Cat. No. #80311) according to a previously described protocol [41]. Briefly, samples were transferred to a QIAGEN PowerBead Plate containing 650 μL of lysis buffer RLT + β-mercaptoethanol and mechanically lysed using a QIAGEN TissueLyzer II (Cat. No. #85300). The lysate was then transferred to the AllPrep 96 DNA Plate and the QIAGEN protocol for “Simultaneous Purification of DNA and RNA from Tissues Using Spin Technology” was followed beginning at step 5. For positive and negative DNA extraction controls, mock microbial communities and molecular-grade water were carried through the entire protocol, respectively.

### Bacterial 16S-V4 rRNA gene sequencing

Bacterial 16S-V4 rRNA gene sequencing libraries were created as previously described [41, 65]. Briefly, PCR amplification of the 16S rRNA gene was conducted in triplicate for each sample using barcoded primers targeting the V4 region [65]. Blank extractions were used as a template for negative controls to monitor for 16S rRNA gene contamination. Following PCR, triplicates were pooled, purified, and normalized. All microbiome sequence data obtained from murine experiments is available from the Sequence Read Archive (http://www.ncbi.nlm.nih.gov/sra, BioProject: PRJNA782012). All microbiome sequence data obtained from clinical trial NCT02749630 is available from the European Genome-Phenome Archive (https://ega-archive.org/, EGAS00001006172).

### 16S-V4 rRNA gene sequence data processing

QIIMEv2019.7 was used to process the 16S-V4 rRNA gene sequence data as previously described [41, 66]. Briefly, raw sequence data was demultiplexed, read trimming was performed to remove regions of low sequence quality, and paired-end reads were denoised, dereplicated, and chimera filtered with DADA2 [67]. Taxonomy was assigned based on the V4 region of the Genome Taxonomy Database 16S rRNA gene sequence database (r86.1) [68, 69]. Resulting in abundance tables were rarefied to 49,000 reads per sample.

### PICRUSt2

Phylogenetic Investigation of Communities by Reconstruction of Unobserved States 2 (PICRUSt2) was used to predict microbiota functional gene abundances from 16S-V4 rRNA gene sequence data [34]. Counts of Enzyme Commission Numbers (EC) were predicted from rarefied abundance tables and representative sequences with the picrust2_pipeline.py function, accounting for the predicted 16S rRNA gene copy number.

### Microbiome analysis

⍺- and β-diversity measures were calculated in QIIMEv2019.7. All statistical analyses were conducted in the R statistical environment [70]. A Wilcoxon rank-sum test was performed to compare ⍺-diversity between experimental groups differences (e.g., aRW vs. IL-22Fc). Bray-Curtis distance matrices were visualized via NMDS and permutational multivariate analysis of variance (PERMANOVA) was used to determine relationships between metadata (i.e., experimental group) and bacterial microbiota composition in the R statistical environment using the vegan package [71]. To identify significantly enriched or depleted bacterial genera, the DESeq2 R-package was used as described by McMurdie et al. and implemented by Nearing et al. [29, 31, 72]. Significant genera were defined as those having a Benjamini-Hochberg adjusted *p* value <0.05 [73]. Only genera with at least 10 reads detected in at least 20% of samples were considered for differential abundance testing. Nonparametric differential abundance testing was performed by subjecting rarefied count data to a Wilcoxon rank-sum test followed by Benjamini-Hochberg *p* value correction, as implemented by Nearing et al. [31]. Compositional data analysis for differential abundance testing was conducted using ALDEx2 as implemented by Nearing et al. [30, 31]. A Wilcoxon rank-sum test was performed to identify differentially enriched predicted metagenomic functions.

### Bacterial strains and growth condition

Bacterial strains were obtained from Deutsche Sammlung von Mikroorganismen und Zellkulturen (DSMZ), Japan Collection of Microorganisms (JCM), Biodefense and Emerging Infections Research Resources Repository (BEI Resources), or isolated in the house (Table S4). A starter culture for each strain was grown in the indicated growth media in a Coy anaerobic chamber (Coy Laboratory) at 37°C for 72 h. Aliquots of the starter culture were then normalized to 0.05 OD600 in 5 mL of fresh growth media. Cultures grown in Chopped Meat Medium with Carbohydrates (CMC, Anaerobe Systems Catalog #AS-823), as well as sterile, negative control CMC, were filtered through a 100-μm cell strainer prior to OD600 measurement. Following normalization, cultures were incubated at 37°C for 48 h, after which cultures were centrifuged at 14,000 rpm for 10 min, filtered using a 0.2-μm syringe filter, and the resulting filtered supernatants were stored at −80°C until use for assessing in vitro AhR activation potential and metabolomics.

### Bacterial genome analysis

TBLASTN 2.11.0+ [74] was used to compare TnaA [*E. coli*], TrpA [*E. coli*], TrpB [*E. coli*],TDC [*Staphylococcus epidermidis*], DDC [*Bacillus licheniformis*], IpdC [*Azospirillum*], AofH [Bacillus subtilis], and AldA [*E. coli*] protein sequences to publicly available sequenced bacterial genomes for isolates GNE6609, GNE6603, GNE6686, and GNE6624 (Genbank: GCF_000011065.1, GCF_900106755.1, GCA_000177015.3, and GCA_003202955.1). Searches yielding high*-*scoring segment pairs (HSPs) satisfying *e* value ≤ 0.1 and query coverage ≥ 60% were considered putative evidence of the presence of the query protein in the target genome.

### Metabolomics

A quantitative targeted metabolomics panel-based LC-MS/MS method, which included analysis of Tryptophan metabolism pathway metabolites, was performed. Analysis was also performed by a pre-qualified liquid chromatography-tandem mass spectrometry (LC-MS/MS) quantitation method which includes 20 tryptophan metabolism pathway metabolites (see the list of analytes in the supplementary table S6) and 17 stable isotope-labeled internal standards for quantitation by surrogate matrix (methanol) approach as described previously for other panels [75, 76]. L-Trp metabolism panel sample preparation includes protein precipitation using organic solvent methanol containing stable isotope-labeled internal standards for optimal recovery. Isomeric or closest eluting stable isotope-labeled internal standards were used for analytes with no corresponding stable isotope-labeled internal standards. Sample batch analysis included surrogate matrix calibration curves for each analyte, and batch performance was evaluated based on the accuracy and precision of quality control samples such as sample pool quality control samples, surrogate matrix quality control samples, and calibration curve samples. After satisfactory sample batch analysis, concentration data for each metabolite was reported in ng/g or μg/g (nM or μM) for fecal samples in a predetermined standardized data format. See Supplementary Table S6 for list of panel analytes, stable isotope-labeled internal standards, and additional protocol details.

### AhR activity assay

AhR activity reporter cell line H1L6.1c3 was obtained from the Denison lab at UC Davis and utilized to assess the AhR activation potential of fecal samples, metabolites, and bacterial culture supernatants according to previously published protocols [37]. In brief, fecal samples were prepared by resuspending fecal pellets in sterile PBS to a final concentration of 100 mg/mL, centrifuging at 4°C to pellet particulate matter, and filtering the supernatant through a sterile 0.22-uM cellulose filter. Prepared fecal samples were stored at −80°C until needed. H1L6.1c3 cells were treated with 100uL of prepared fecal samples diluted 1:10 in growth media [Alpha-Minimal Essential Media (MEM; Invitrogen, #12000-063) containing 10% fetal bovine serum] and incubated at 33°C in the presence of 5% CO_2_ for 24 h, afterwhich the luciferase activity was measured. For bacterial culture supernatants, H1L6.1c3 cells were treated with 100uL of sterile-filtered bacterial culture supernatant diluted 1:10 in growth media [Alpha-Minimal Essential Media (MEM; Invitrogen, #12000-063) containing 10% fetal bovine serum] and incubated at 33°C in the presence of 5% CO_2_ for 24 h, after which luciferase activity was measured. For IL-22Fc responsive fecal metabolites (L-Trp, I3C, and 5-HIAA), H1L6.1c3 cells were treated with 100uL of growth media [Alpha-Minimal Essential Media (MEM; Invitrogen, #12000-063) containing 10% fetal bovine serum] containing 100uM of either L-Trp, I3C, or 5-HIAA and incubated at 33°C in the presence of 5% CO_2_ for 4 h, afterwhich the luciferase activity was measured metabolites. All samples were measured in triplicate. Statistically significant differences in AhR activation potential were determined by Wilcoxon rank-sum test.

### IL-22Fc Ph1b study protocol

Healthy volunteers and UC patients with moderate to severe ulcerative colitis were enrolled in a phase 1b multicenter, randomized, observer-blinded, placebo-controlled study to evaluate the safety, tolerability, pharmacokinetics, and pharmacodynamics of efmarodocokin alfa [41]. Briefly, 69 HV and 46 UC patients screened for this study provided baseline stool samples. Twenty-four UC patients met the study enrollment criteria (including diagnosis of UC with a Mayo Endoscopic Subscore of ≥2 points by central reading at screening) and were administered placebo (*n*=6) or efmarodocokin alfa (*n*=18) intravenously at doses ranging from 30 to 90 μg/kg either biweekly or monthly. Stool samples were obtained predose and at defined post-dose time points, with missing timepoints treated as absent. A total of five placebo-treated UC patients and nine efmarodocokin alfa-treated UC patients had complete stool sample collection history for the entire study (screening, day 29, day 43, day 64, and day 85). Note that fecal samples for one efmarodocokin alfa-treated UC patient were exhausted prior to performing the in vitro AhR activity assay, leading to *n*=5 placebo-treated UC patients and *n*=8 efmarodocokin alfa-treated UC patients for this analysis.

## Supplementary Information

**Additional file 1: Figure S1.** Supplement to IL-22Fc microbiota response in mice. (A) After a two week acclimatization period during which animals were co-housed, six- to eight-week-old, female C57BL/6J mice (*n*=16) were randomly re-caged on study day 0 into two treatment groups and separately housed for the remainder of the experiment: IL-22Fc treated (*n*=8, four mice per cage) and control (*n*=8, four mice per cage). Animals were treated with 50 ug/mouse of either IL-22Fc or anti-ragweed isotype control via intraperitoneal injection three times a week for two weeks (as indicated by the red arrow), and euthanized on day 14. See methods for additional details. (B) Richness following IL-22Fc or aRW treatment. P-values determined by T-test. (C-D) Normalized abundance in control and IL-22Fc treated animals of the top highly IL-22Fc responsive bacterial genera consistently enriched (C) or depleted (D). Statistical significance determined by DESeq2 and corrected for false discovery. * BH-corrected *p*-value <0.05. For all plots, triangle, square, and circle markers indicate ileal, colonic, and fecal samples respectively. Teal and red markers indicate animals treated with (IL-22Fc (*n*=8) or isotype control (*n*=8), respectively, respectively. **Figure S2.** Tryptophan metabolism pathways. (A) Depiction of well established tryptophan metabolism pathways relevant to the GI microbiome. As the functional capacity of the gut microbiome is highly complex and yet to be fully characterized, additional, as yet unidentified microbial metabolic pathways involving L-Trp metabolism may exist. Predominantly microbiome derived enzymes and metabolites are highlighted in green [13, 20]. L-Trp: L-Tryptophan, 5-HT: Serotonin, 5-HIAAld: 5-Hydroxyindoleacetaldehyde, 5-HIAA: 5-Hydroxyindoleacetic acid, L-Kyn: L-Kynurenine, ILA: Indole Lactic Acid, IPA: Indole Propionic Acid, IAAld: Indole Acetaldehyde, IAA: Indole Acetic Acid, I3C: Indole-3-Carboxaldehyde. **Figure S3.** Supplement to IL-22Fc tryptophan metabolism response in mice. (A) Fecal concentrations of additional microbially derived L-Trp metabolites included in our panel that were not significantly altered by IL-22Fc treatment. Teal and red markers indicate animals treated with IL-22Fc (*n*=8) or isotype control (*n*=8), respectively. (B) Log_2_(Fold change from baseline) in fecal concentrations of additional microbially derived L-Trp metabolites included in our panel. Statistical significance determined by Rank-Sum Test (. *p*<0.1, * *p*<0.05, ** *p*<0.01). (C) Predicted prevalence of tryptophan synthase, tryptophanase, and aldehyde dehydrogenase among ASVs representing the highly IL-22Fc enriched or depleted bacterial genera. Statistical significance determined by Barnard’s Test (* *p*<0.05). (D) Predicted prevalence of tryptophan synthase, tryptophanase, and aldehyde dehydrogenase among ASVs representing the highly IL-22Fc enriched or depleted bacterial genera, stratified by genus. Teal and Red bars indicate bacterial genera enriched or depleted following IL-22Fc treatment, respectively. **Figure S4.** Supplement to Intestinal AhR Signaling following IL-22Fc treatment in mice. (A) Scatterplot of L-Trp, I3C, and 5-HIAA fecal concentration vs. fecal AhR activity. (B-C) Scatterplot of predicted ileal (B) or colonic (C) metagenomic counts of tryptophan synthase, tryptophanase, and aldehyde dehydrogenase vs. fecal AhR activity. For all panels, Spearman's rank correlation coefficient (rho) was calculated to determine correlation strength. **Figure S5.** Supplement to Proxy Strain AhR Activity and Metabolite Production. (A-B) AhR activity, reported as Log2(fold change over matched sterile culture media) of sterile filtered culture supernatant from bacterial isolates representing the highly IL-22Fc depleted (A) or enriched (B) genera, stratified by bacterial isolate. (C) Metabolite production, reported as Log2(fold change over matched sterile culture media) of sterile filtered culture supernatant from bacterial isolates that induced AhR activity, stratified by bacterial isolate. Culture supernatants were collected and measured in triplicate for all panels. Statistical significance indicates relative AhR activity or metabolite concentration is statistically greater than zero as tested by one sample T-test (* *p*<0.05, ** *p*<0.01), indicating an *in vitro* AhR activity or metabolite concentration is statistically greater than that of matched sterile culture media. **Figure S6.** Supplement to efmarodocokin alfa effect on fecal metabolite concentrations in humans. (A) Fecal concentrations of L-Kyn at screening in HV (*n*=69) compared to UC (*n*=46). P-value determined by Rank-Sum test. (B) Fecal AhR Activity (reported as fold change over unstimulated wells) at screening in HV (*n*=69) and UC (*n*=46). P-value determined by T-test. (C) Fecal concentrations of L-Trp, I3C, IPA, and L-Kyn at screening in HV (*n*=69), moderate UC (mMCS ≤ 5, *n*=10), and severe UC (mMCS > 5, *n*=13). P-value determined by Rank-Sum test. (D) Fecal AhR Activity (reported as fold change over unstimulated wells) at screening in HV (*n*=69) moderate UC (mMCS ≤ 5, *n*=10), and severe UC (mMCS > 5, *n*=13). P-value determined by T-test. (E) AUC of Log_2_(fold change from screening) for fecal concentrations of L-Trp, I3C, IPA, and L-Kyn in enrolled UC participants following administration of efmarodocokin alfa (*n*=9) or placebo (*n*=5). P-value determined by T-Test. For panel E, only enrolled UC participants with a complete sample collection history (screening, day 29, day 43, day 64, and day 85) were considered for AUC calculations. **Figure S7.** Supplement to efmarodocokin alfa effect on fecal microbiota in enrolled trial participants. (A) Mean Log_2_(fold change in abundance at screening between enrolled UC and HV) of the IL-22Fc depleted (*n*=8) and IL-22Fc enriched (*n*=5) genera present in the fecal microbiome of enrolled trial participants at screening. P-value determined by T-test. (B) Normalized abundance of highly IL-22Fc depleted (B) or enriched (C) genera in the fecal microbiome of enrolled HV (*n*=39) and UC (*n*=24) participants at screening. P-value determined by DESeq2 analysis. **Figure S8.** Supplement to efmarodocokin alfa effect on fecal microbiota AUC in humans. AUC of Log_2_(fold change from screening) for normalized abundance of IL-22Fc depleted (A) or enriched (B) genera detected in enrolled UC patients receiving either placebo (*n*=5) or efmarodocokin alfa (*n*=9) treatment. Only enrolled UC patients with a complete sample collection history (screening, day 29, day 43, day 64, and day 85) were considered for AUC calculations. Note that *CAG-873* was not detected in any enrolled UC patients with a complete sampling history. P-value determined by T-Test.

**Additional file 2: Table S1-3.** Differential Abundance Testing Results. To identify significantly enriched or depleted bacterial genera in the (S1) ileum, (S2) colon, and (S3) stool of mice treated with IL-22Fc compared to placebo, the DESeq2 R-package was used as described by McMurdie et al. [72]. We performed two additional, conceptually different statistical approaches for DA testing, nonparametric Wilcoxon rank-sum testing as well as compositional data analysis utilizing ALDEx2 to ensure our observations were robust to the statistical method implemented. Effect sizes, nominal p-values, and BH-adjusted p-values for all statistical approaches are displayed. Only genera with at least 10 reads detected in at least 20% of samples were considered for differential abundance testing. **Table S4.** Bacterial Strain Information. Strain ID, source, strain name, whole genome and 16S rRNA gene sequence accession number, preferred growth medium, and proxy taxonomy (i.e. experimentally identified bacterial taxa this strain is representing based on ≥97% 16S rRNA gene sequence similarity) for all bacterial strains used in this study. All strains sourced from Genentech (i.e.: Source = ‘Genentech’) were isolated directly from mice utilized in the described experiments while all other strains were acquired from the indicated culture collection. **Table S5.** Evidence for the capacity to produce tryptophan derived AhR ligands in sequenced genomes of putative AhR activating bacterial strains. Shown are the results of genome-wide BLAST comparisons of TnaA [E. coli], TrpA [E. coli], TrpB [E. coli], TDC [Staphylococcus epidermidis], DDC [Bacillus licheniformis], IpdC [Azospirillum], AofH [Bacillus subtilis] and AldA [E. coli] protein query sequences against sequenced genomes of bacterial strains GNE6609, GNE6603, GNE6686, and GNE6624. Each line within a field represents a high-scoring segment pair (HSP) yielded by tBLASTn searches of query protein sequences against the corresponding translated bacterial isolate genome with evalue ≤ 0.1. Genes are considered putatively present if BLAST searches yield at least one HSP satisfying query coverage ≥ 60%. **Table S6.** List of metabolomics panel analytes, stable isotope labeled internal standards, and additional protocol details.

## Data Availability

The 16S-V4 rRNA gene sequencing dataset analyzed in this study is publicly available from the Sequence Read Archive (SRA) database under the BioProject accession number PRJNA782012. Additional data related to this research may be requested from the authors.

## Abbreviations

AhR: Aryl hydrocarbon receptor
L-Trp: L-Tryptophan
UC: Ulcerative colitis
ILC3: Type 3 innate lymphoid cells
GI: Gastrointestinal
IEC: Intestinal epithelial cells
AMP: Antimicrobial peptide
SCFA: Short chain fatty acid
Treg: Regulatory T cell
IL-22Fc: Fusion protein of IL-22 and the Fc portion of IgG4
aRW: Anti-ragweed isotype control antibody
I3C: Indole-3-carboxaldehyde
5-HIAA: 5-Hydroxyindoleacetate
IPA: Indole-3-propionic acid
IAA: Indole-3-acetic acid
ASV: Amplicon sequence variant
L-Kyn: L-Kynurenine
AUC: Area under the curve
GVHD: Graft-versus-host disease
