# Supplementary figures and images for: IL-22 alters gut microbiota composition and function to increase aryl hydrocarbon receptor activity in mice and humans
Source: Microbiome. 2023 Mar 9;11:47. doi: 10.1186/s40168-023-01486-1 (PMC9997005; doi:10.1186/s40168-023-01486-1)

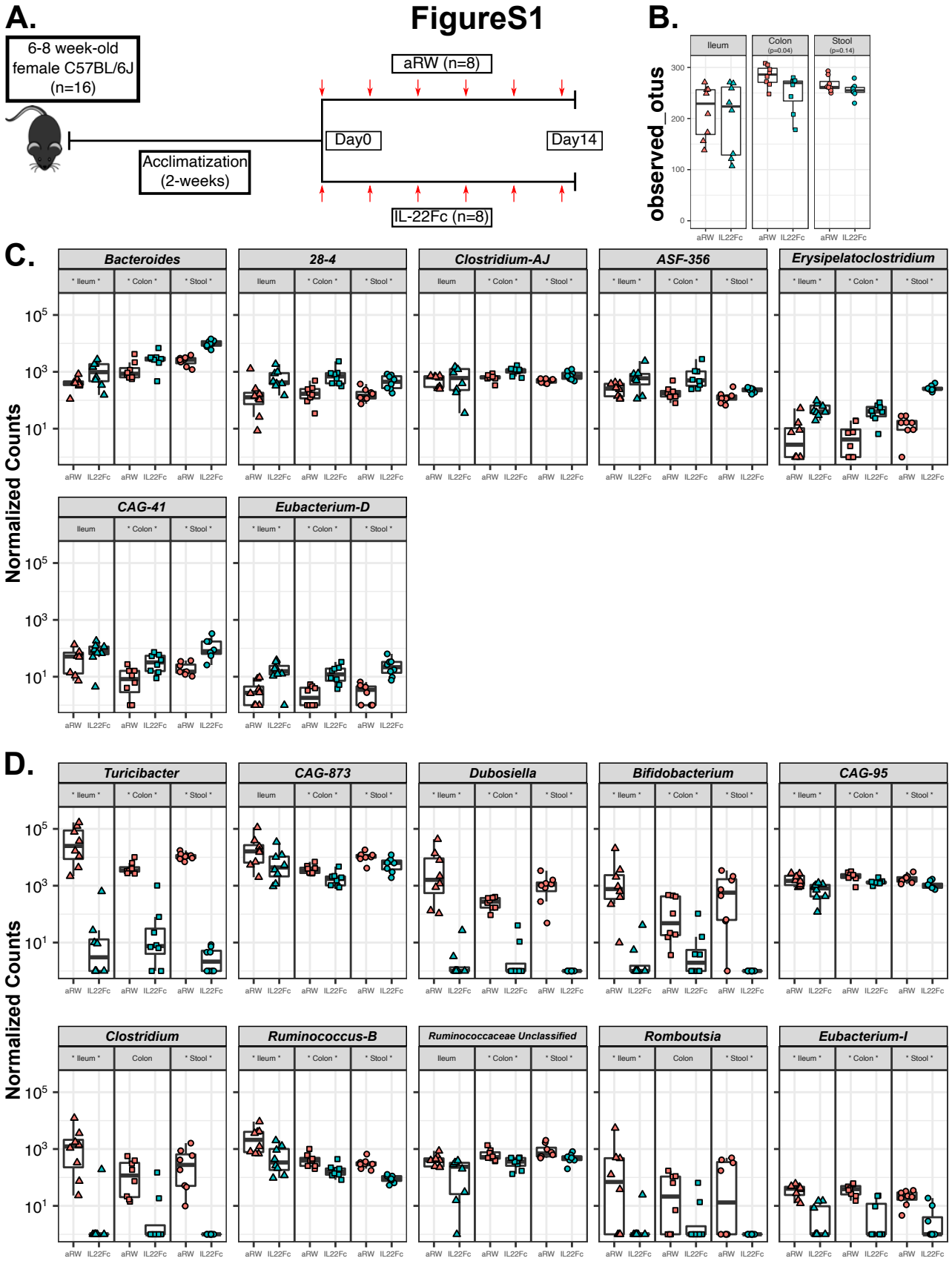

Figure S2

A.

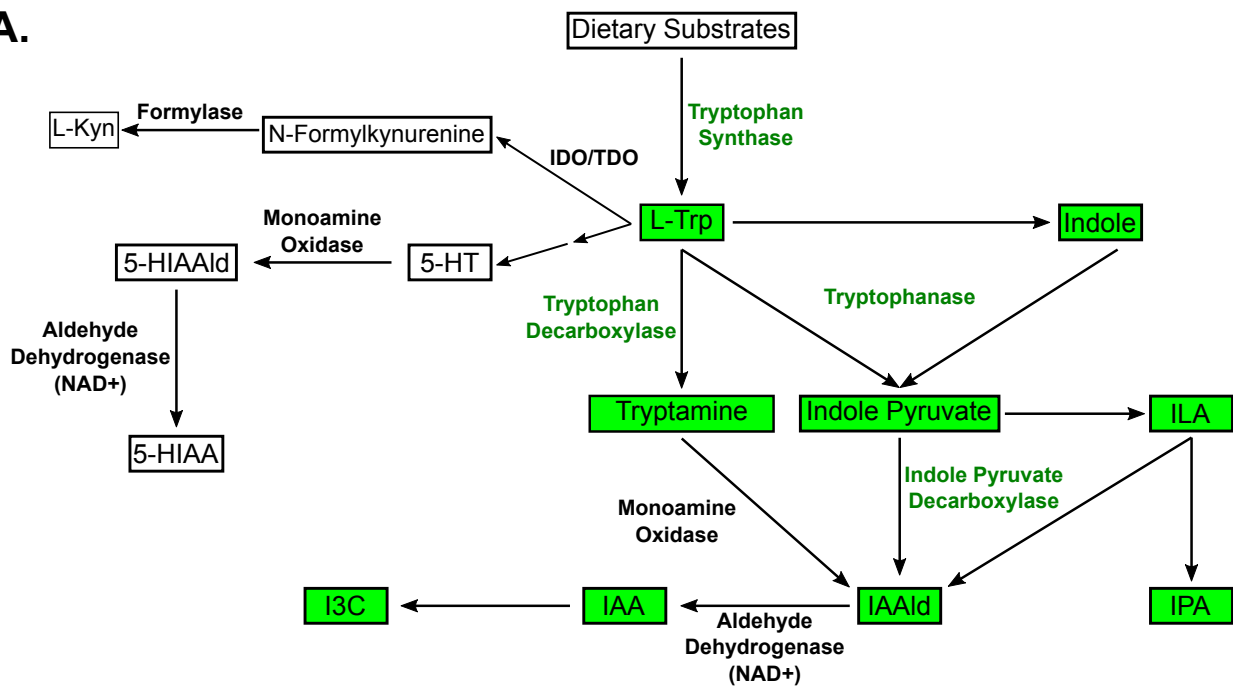

# Figure S3

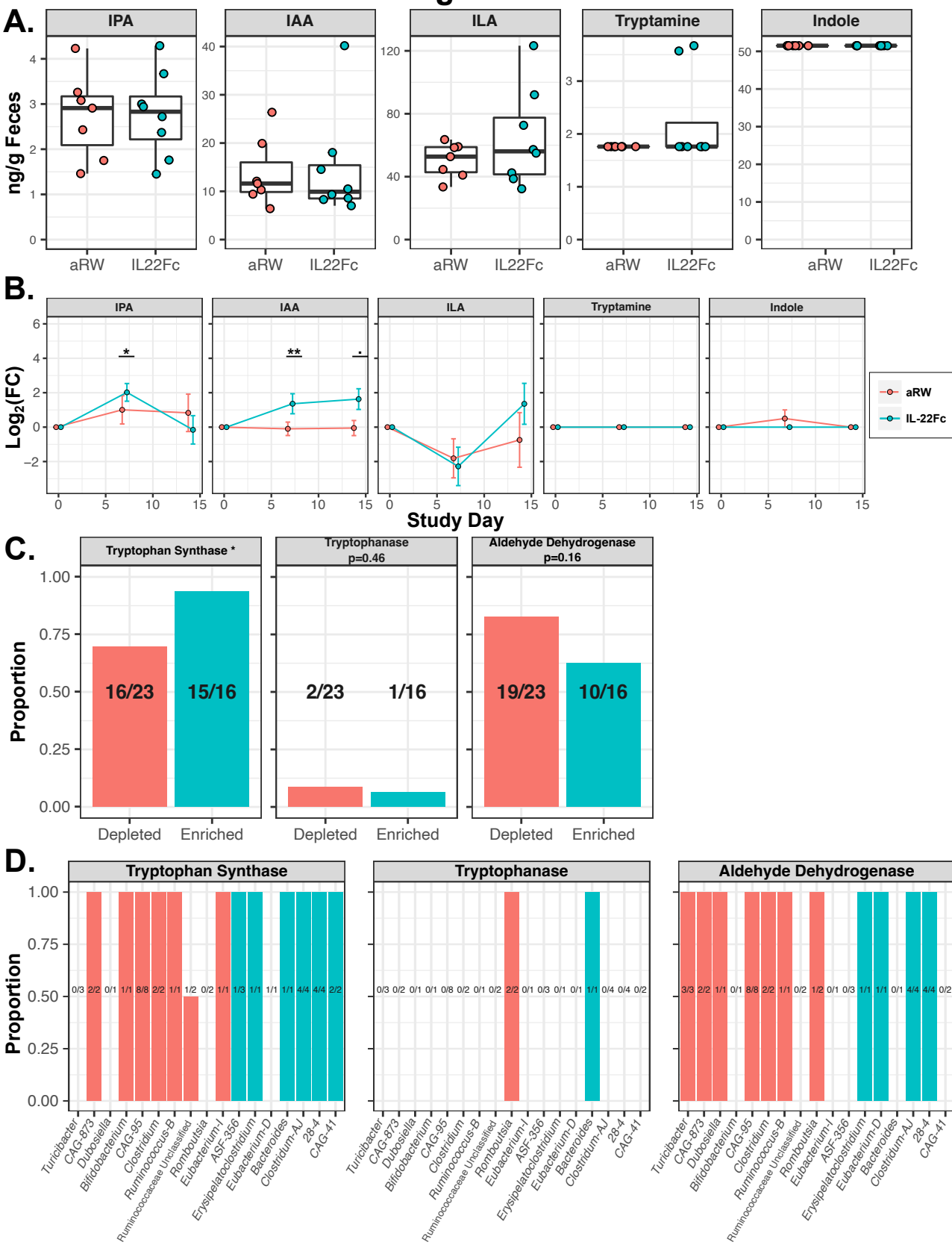

FigureS4

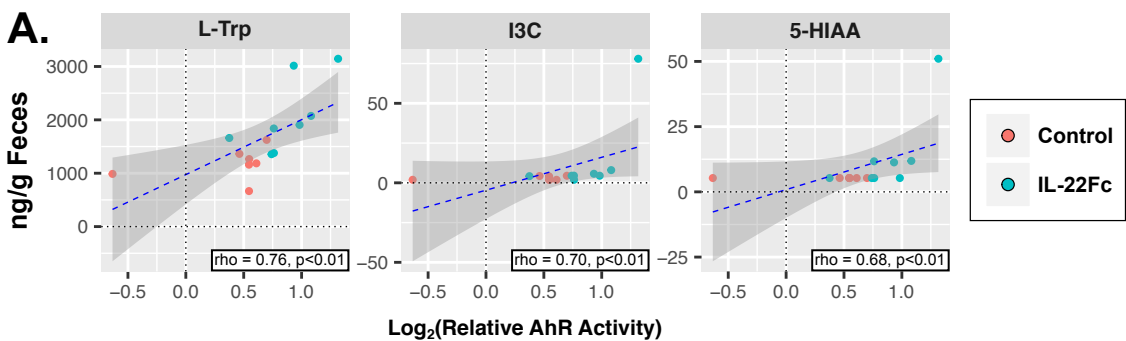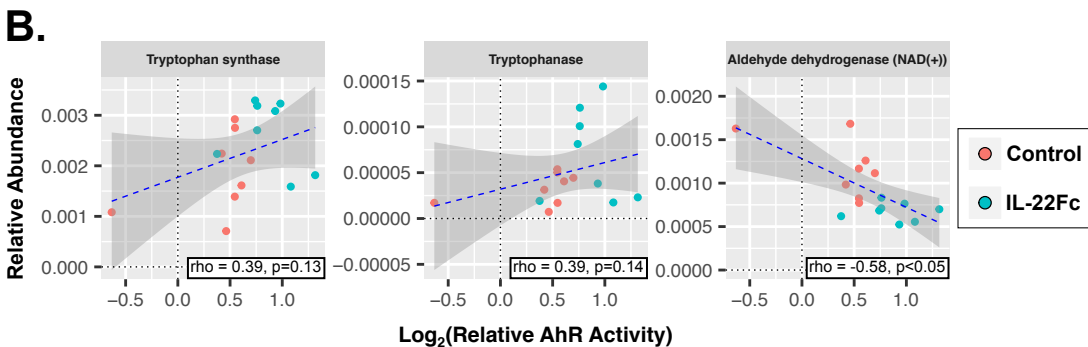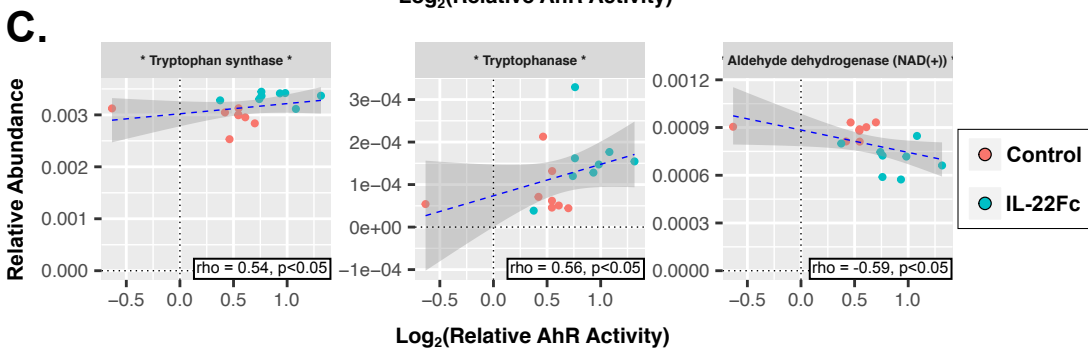

FigureS5

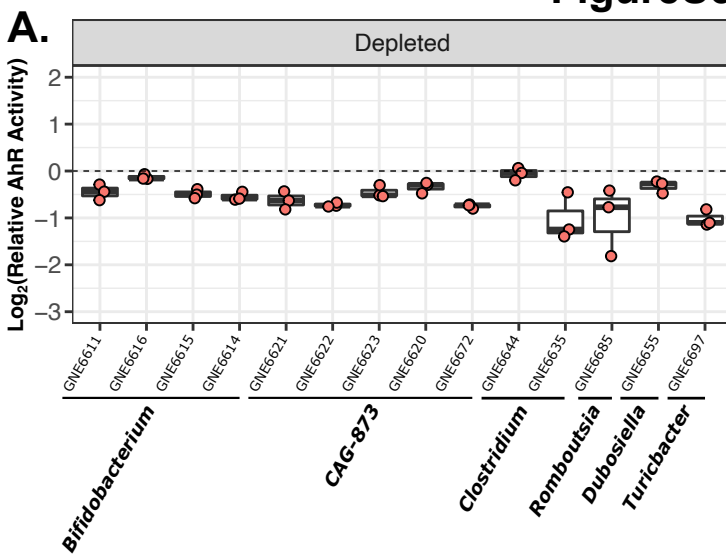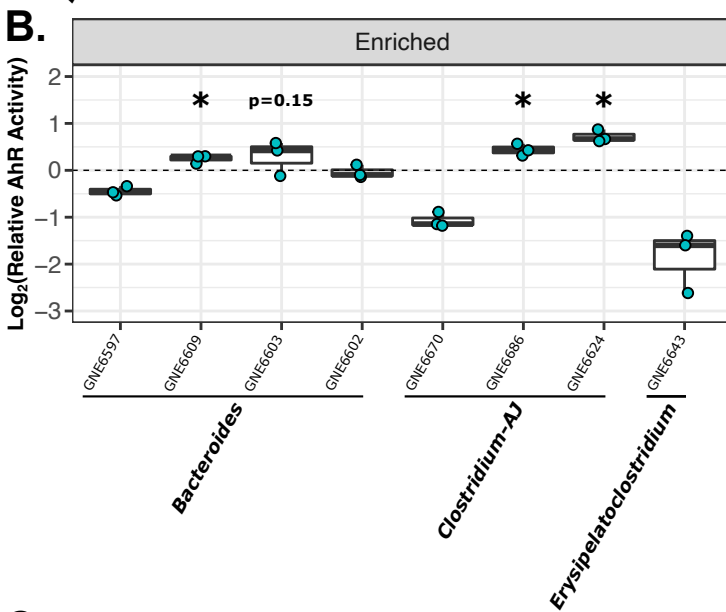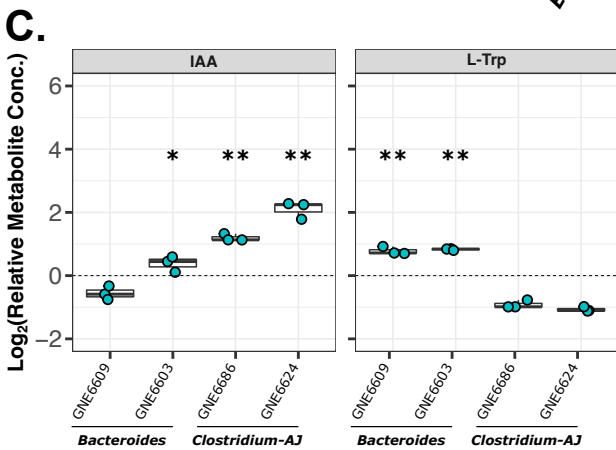

**FigureS6**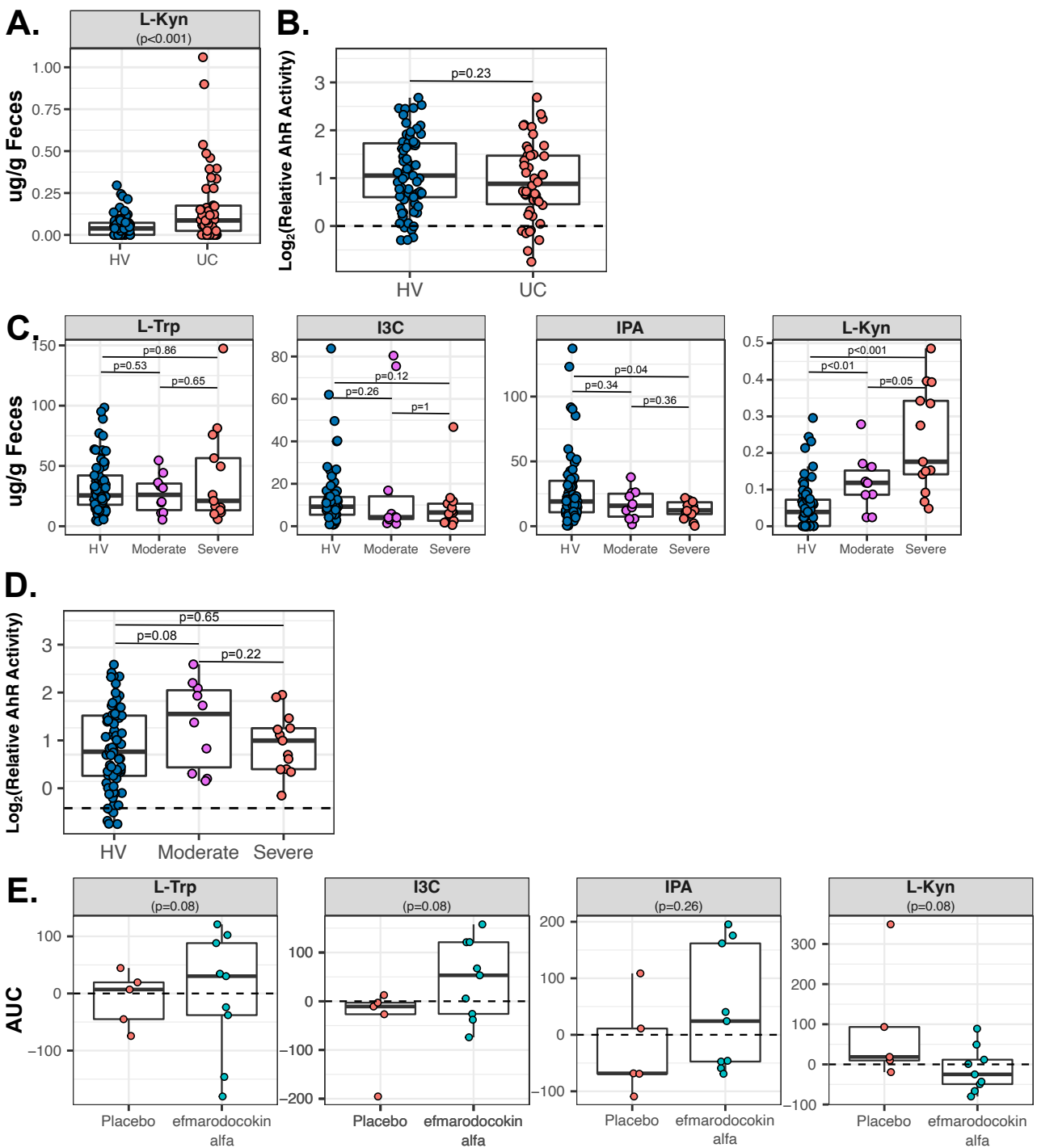

# FigureS7

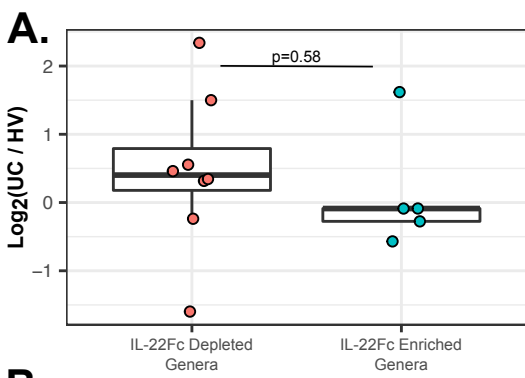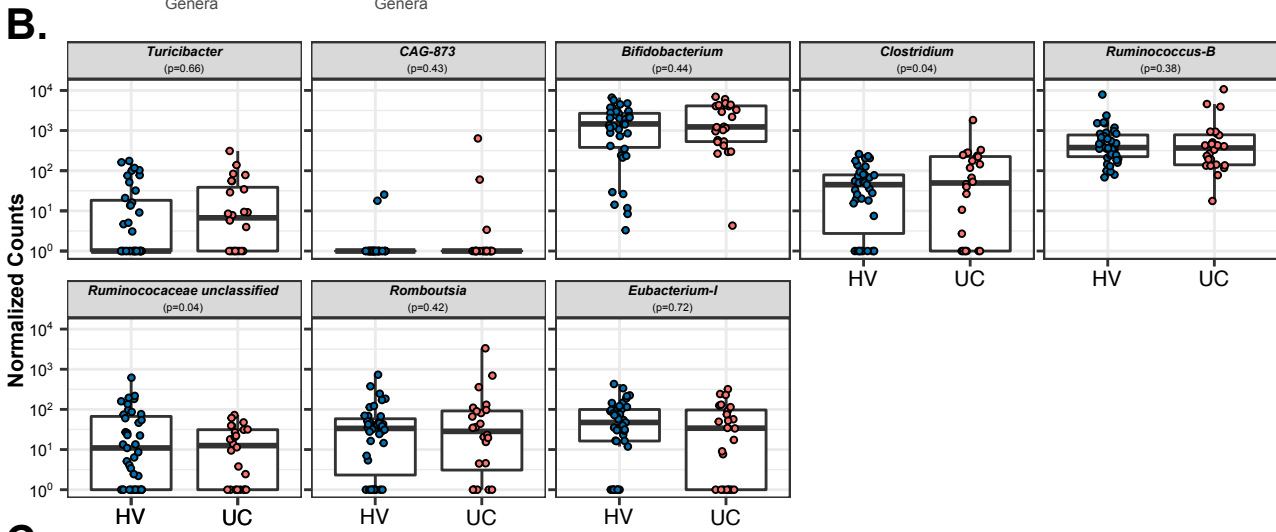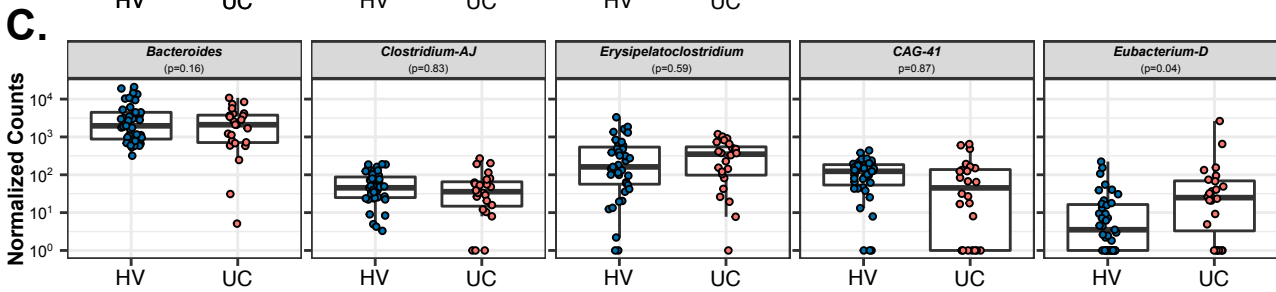

# FigureS8

**A.**

AUC

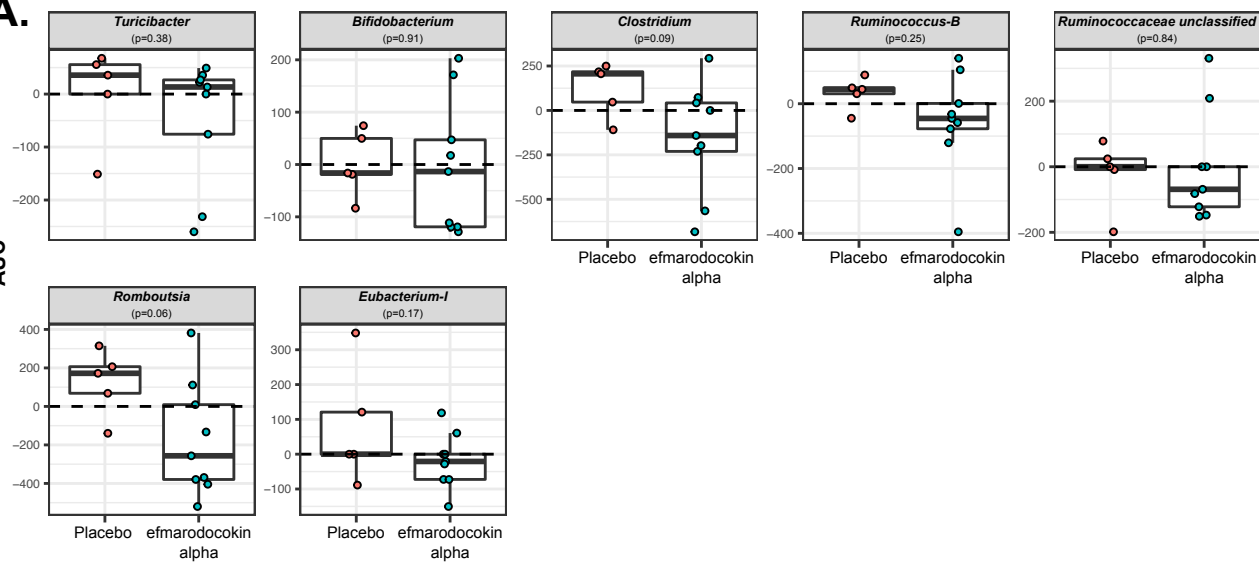

**B.**

AUC

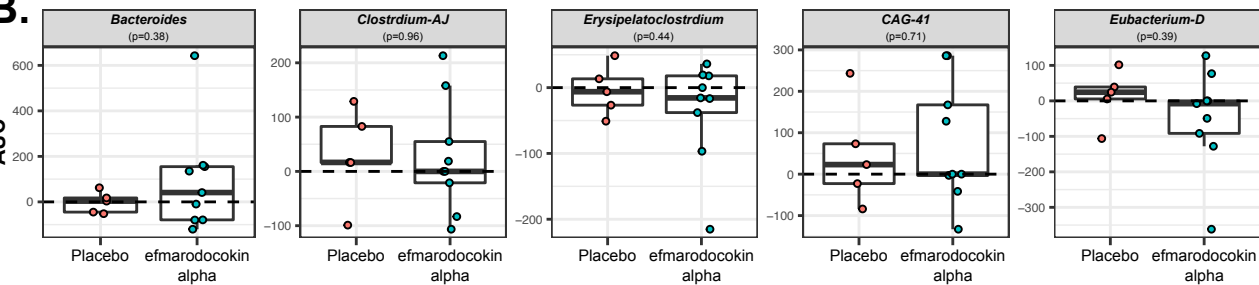

Supplement: Supplementary file 2 — Additional file 1: Figure S1. Supplement to IL-22Fc microbiota response in mice. (A) After a two week acclimatization period during which animals were co-housed, six- to eight-week-old, female C57BL/6J mice (n=16) were randomly re-caged on study day 0 into two treatment groups and separately housed for the remainder of the experiment: IL-22Fc treated (n=8, four mice per cage) and control (n=8, four mice per cage). Animals were treated with 50 ug/mouse of either IL-22Fc or anti-ragweed isotype control via intraperitoneal injection three times a week for two weeks (as indicated by the red arrow), and euthanized on day 14. See methods for additional details. (B) Richness following IL-22Fc or aRW treatment. P-values determined by T-test. (C-D) Normalized abundance in control and IL-22Fc treated animals of the top highly IL-22Fc responsive bacterial genera consistently enriched (C) or depleted (D). Statistical significance determined by DESeq2 and corrected for false discovery. * BH-corrected p-value <0.05. For all plots, triangle, square, and circle markers indicate ileal, colonic, and fecal samples respectively. Teal and red markers indicate animals treated with (IL-22Fc (n=8) or isotype control (n=8), respectively, respectively. Figure S2. Tryptophan metabolism pathways. (A) Depiction of well established tryptophan metabolism pathways relevant to the GI microbiome. As the functional capacity of the gut microbiome is highly complex and yet to be fully characterized, additional, as yet unidentified microbial metabolic pathways involving L-Trp metabolism may exist. Predominantly microbiome derived enzymes and metabolites are highlighted in green [13, 20]. L-Trp: L-Tryptophan, 5-HT: Serotonin, 5-HIAAld: 5-Hydroxyindoleacetaldehyde, 5-HIAA: 5-Hydroxyindoleacetic acid, L-Kyn: L-Kynurenine, ILA: Indole Lactic Acid, IPA: Indole Propionic Acid, IAAld: Indole Acetaldehyde, IAA: Indole Acetic Acid, I3C: Indole-3-Carboxaldehyde. Figure S3. Supplement to IL-22Fc tryptophan metabol [file 40168_2023_1486_MOESM1_ESM.pdf]
